# A Case of Unusual Vascularization of Upper Abdominal Cavity' Organs

**DOI:** 10.1155/2018/5738037

**Published:** 2018-10-31

**Authors:** Natalia Mazuruc, Serghei Covantev, Olga Belic

**Affiliations:** ^1^Department of Human Anatomy, State University of Medicine and Pharmacy «Nicolae Testemitanu», Chisinau, Moldova; ^2^Laboratory of Allergology and Clinical Immunology, State University of Medicine and Pharmacy «Nicolae Testemitanu», Chisinau, Moldova

## Abstract

We describe a case report of multiple arterial variations of internal organs of upper abdominal cavity in a cadaver of 63-year-old female. There were several developmental variations of the vascular supply of the stomach, pancreas, spleen, and liver. There were several accessory arteries: left gastric, left hepatic, and posterior gastric artery as well as several arteries that had abnormal origin. The variations were discovered during macroscopical dissection at the department of human anatomy. It should be noted that multiple developmental variation can be common in clinical practice and clinicians should be aware of them during diagnostic and interventional procedures.

## 1. Introduction

The upper part of abdominal cavity is limited by the diaphragm from the top, from the sides by lateral abdominal walls and from the inferior part by the transverse colon and its mesentery. This anatomical region is particularly important for hepatopancreatobiliary, vascular, and transplant surgeons as it is rich in anatomical variations that can compromise the procedure. The vascularization of upper abdominal cavity' organs has been studied abundantly [1].

The celiac trunk provides the vascular supply of the upper abdominal organs. Uflacker classified the celiac trunk into 8 types: classic coeliac trunk, hepatosplenic trunk, hepatogastric trunk, hepatosplenicmesenteric trunk, gastrosplenic trunk, coeliac-mesenteric trunk, and no coeliac trunk [2]. Michel classified the vascular supply of the liver in 10 types and later Hiatt modified this classification [3, 4]. The splenic artery may have two, three, four, and five terminal branches or enter the splenic tissue without branching. A more complex classification is provided by Vandamme and Bonte who describe bifurcation of the splenic artery into two rami lienalis where the gastroepiploic artery is a collateral of the splenic stem; trifurcation of the splenic artery; truncus lienogastroepiploicus [5].

Detailed knowledge of topographical and morphological as well as the functional possibilities of the anatomical regional and its surrounding structures including collateral paths of vascularization is essential for surgical procedures [6]. It is important to limit the risk of vascular injury, particularly in the presence of anatomical variations for a variety of procedures and especially in transplant surgery [7].

## 2. Anatomical Case

During the macroscopical dissection of a 63-year-old female cadaver we determined several anomalies of the vascularization of upper abdominal cavity organs.

**Figure 1:**
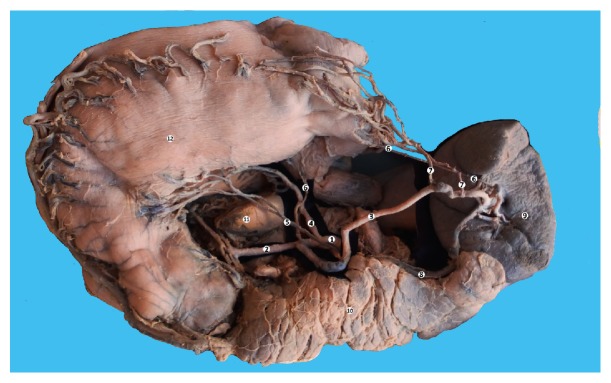
The branches of the celiac trunk. Macrospecimen (female, 63 years old). 1: celiac trunk, 2: common hepatic artery, 3: splenic artery, 4: left gastric artery, 5: accessory left gastric artery, 6: posterior gastric artery, 7: left gastroepiploic artery 8: splenic vein, 9: spleen, 10: pancreas, 11: caudate lobe of the liver, and 12: stomach.

The celiac trunk branched into two vessels: the left gastric artery and a hepatosplenic trunk, which further divided into splenic and common hepatic arteries (Figure 1).

The splenic artery in its proximal part had a sinuous trajectory. In the medium part of the vessel the artery had a curve with the base situated superiorly which then continued in a straight manner in the prehilar area. In the hilar region the splenic artery branches into three branches of the first order, two of which were terminal and entered directly into splenic parenchyma.

**Figure 2:**
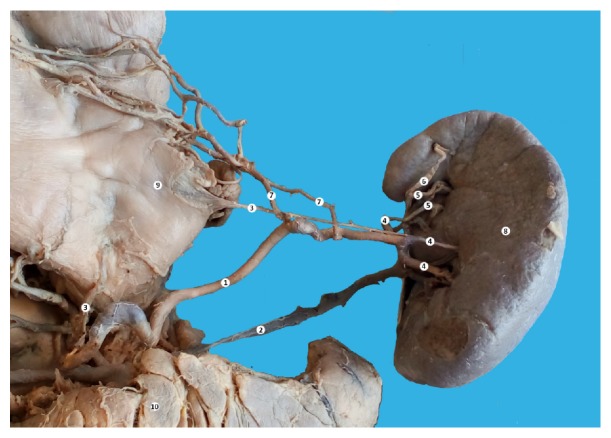
The branches of splenic artery. Macrospecimen (female, 63 years old). 1: splenic artery, 2: splenic vein, 3: posterior gastric artery, 4: splenic arteries of the first order 5: splenic arteries of the second order, 6: superior polar artery 7: left gastroepiploic artery 8: spleen, 9: stomach, and 10: pancreas.

The superior splenic branch of the first order divided into two second order branches. From the superior second order branch a superior polar branch took origin (Figure 2). From the distal third of the splenic artery took origin two left gastroepiploic arteries (at 8 and 10 cm, respectively) which participated in the vascularization of the posterior stomach wall and the great omentum.

We should also mention the presence of two posterior gastric arteries, which also branched from the splenic artery. The first posterior gastric artery took origin from the site, where the splenic artery branched from the hepatosplenic trunk and passed to the fundus of the stomach. The second posterior gastric artery is a more common variant that took origin from the second branch of the first order (Figures 1 and 2). The lesser curve of the stomach was vascularized by two left gastric arteries and right gastric artery. The accessory left gastric artery branched from the proximal third of the splenic artery (2,7 cm from its origin).

**Figure 3:**
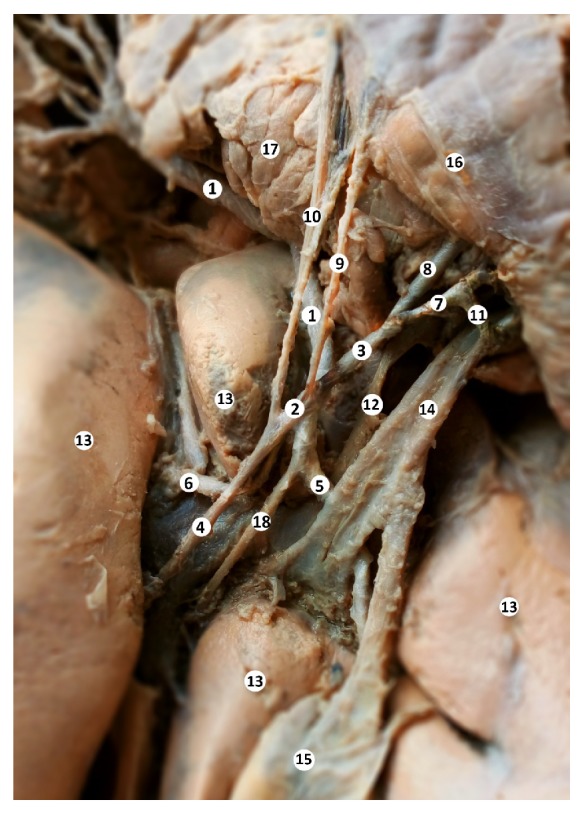
Liver blood supply. Macrospecimen (female, 63-year-old). 1 – common hepatic artery, 2 – proper hepatic artery, 3 – gastroduodenal artery, 4 – left hepatic artery, 5 – right hepatic artery, 6 – accessory left hepatic artery, 7 – right gastroepiploic artery, 8 – superior posterior pancreaticoduodenal artery, 9 – superior duodenal artery, 10 – right gastric artery, 11 – the branch of the superior anterior pancreaticoduodenal artery, 12 – portal vein, 13 – liver, 14 – common bile duct, 15 – gall bladder, 16 – duodenum, 17 – pancreas, 18 – intermediate hepatic artery.

The common hepatic artery passed near the right side of the caudate lobe of the liver and at the level of the pancreas head (after giving a gastroduodenal branch) continued into proper hepatic artery which branched into left hepatic arteries and a larger right hepatic artery (Figure 3).

A rare variation is also the presence of an accessory left hepatic artery, which began from the proper hepatic artery and supplied the left and the caudate lobes of the liver. From the proper hepatic artery took origin the superior duodenal artery that passed to the bulb of the duodenum. The proper hepatic artery then gave off its terminal branches: the right and left hepatic arteries. The intermediate hepatic artery branched from the right hepatic artery and passed to the quadrate lobe of the liver (Figure 3).

**Figure 4:**
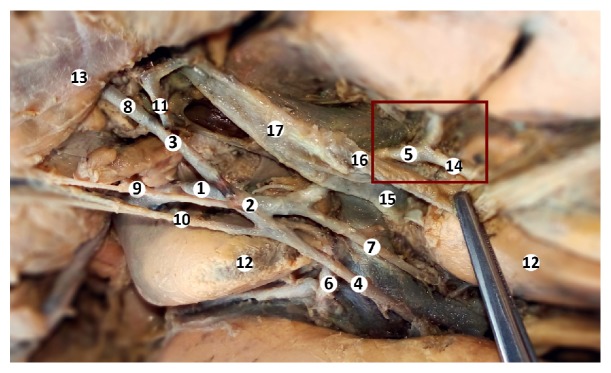
Cystic artery. Macrospecimen (female, 63 years old). 1: common hepatic artery, 2: proper hepatic artery, 3: gastroduodenal artery, 4: left hepatic artery, 5: right hepatic artery, 6: accessory left hepatic artery, 7: intermediate hepatic artery, 8: superior posterior pancreaticoduodenal artery, 9: superior duodenal artery, 10: right gastric artery, 11: right gastroepiploic artery, 12: liver, 13: duodenum, 14: cystic artery, 15: hepatic duct, 16: cystic duct, and 17: common bile duct.

The cystic artery was situated behind the cystic duct, outside the Calot triangle, and had only a posterior branch that entered and vascularized the posterior wall of the gallbladder (Figure 4).

**Figure 5:**
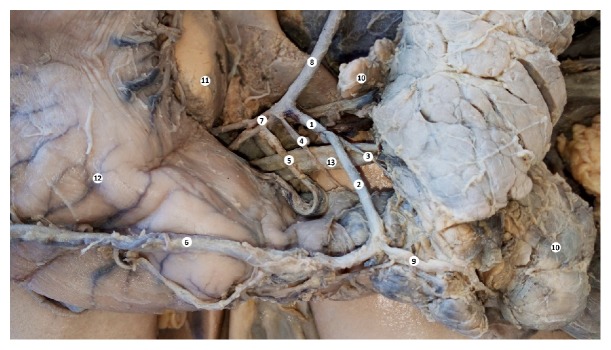
The branches of pancreaticoduodenal artery. Macrospecimen (female, 63 years old). 1: gastroduodenal artery, 2: right gastroepiploic artery, 3: superior posterior pancreaticoduodenal artery, 4: superior duodenal artery, 5: right gastric artery, 6: right gastroepiploic artery, 7: proper hepatic artery, 8: common hepatic artery, 9: superior anterior pancreaticoduodenal artery, 10: pancreas, 11: liver, 12: stomach, and 13: common bile duct.

**Figure 6:**
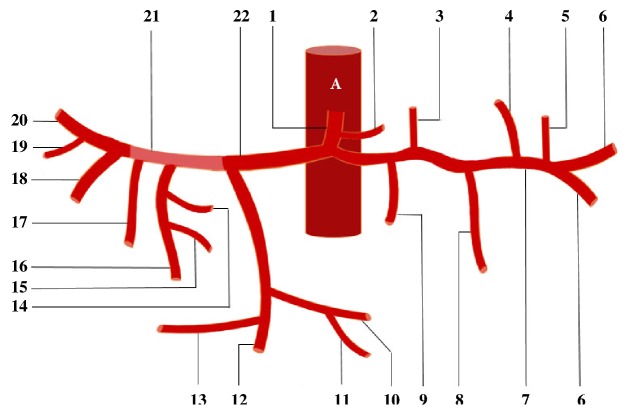
A: abdominal aorta; 1: celiac trunk, 2: left gastric artery, 3: accessory left gastric artery, 4: second left gastroepiploic artery, 5: second posterior gastric artery, 6: terminal branches of the splenic arteries, 7: splenic arteries, 8: left gastroepiploic artery, 9: posterior gastric artery, 10: right gastroepiploic artery, 11: superior anterior pancreaticoduodenal artery, 12: gastroduodenal artery, 13: posterior superior pancreaticoduodenal artery, 14: right gastric artery, 15: superior duodenal artery, 16: left hepatic artery, 17: accessory left hepatic artery, 18: intermediate hepatic artery, 19: cystic artery, 20: right hepatic artery, 21: proper hepatic artery, and 22: common hepatic artery.

The gastroduodenal artery gave two branches: the posterior superior pancreaticoduodenal artery and the right gastroepiploic artery. From the right gastroepiploic artery branched the superior anterior pancreaticoduodenal artery and a short branch to the common bile duct (Figures 3, 4, and 5). A graphical representation of all of the anatomical variations is present in Figure 6.

## 3. Discussions

Based on the current data from the literature there are multiple types of liver and gallbladder vascularization. Watson and Harper (2015) report that the hepatic artery can have variable origin (10 types based on Michels classification, 1955). There are often accessory left and right hepatic arteries (10-20% of cases) [8].

The trifurcation of the hepatic artery can be encountered in approximately 3.3% of cases [9]. The gastroduodenal artery can have the accessory branches or, in rare cases, be absent. In 11% of cases, patients may have an arterial pattern, which is not described in Michel's classification [10]. Although, the presence of accessory hepatic arteries is not uncommon the combination of accessory hepatic arteries and extrahepatic arteries (duodenum and pancreas) is rare. Therefore, the present case demonstrates an even more complicated vascular anatomy at the level of hepatogastric and hepatoduodenal ligament. A vessel is named “additions” or “accessory” typically when the second vessel is smaller in diameter or has an abnormal origin.

De Martino RR and coworkers consider that the anomalies of the splenic artery can be part of the celiac trunk anomalies. The vessel may start from the aorta with one or two branches. It also may take origin from left gastric, middle colic, or left hepatic artery [11]. The present case is an example of hepatosplenic trunk (type II variation by Uflacker). A type II celiac trunk anatomy is found in 7.1-8% of cases [12, 13].

The vascularization of the stomach is also highly variable. The left gastric artery can take origin from aorta, celiac trunk, or accessory left hepatic artery (1-16%) and left hepatic artery (10%) [11] or may have a common trunk with the hepatic artery (1.7%) [9].

Accessory gastric arteries are often present and most commonly originates from hepatic vessels (21.2%) [14]. The right gastric artery more often originates from proper hepatic artery (53%), from the area where the common hepatic artery bifurcates (20%), from left hepatic artery (15%), and less common from gastroduodenal artery (8%) or common hepatic artery (4%) [15]. The present case is uncommon since the lesser curve of the stomach was vascularized by two left gastric arteries and right gastric artery. The accessory left gastric artery branched from the proximal third of the splenic artery. This is a rare case and to our knowledge not previously reported in the literature.

The posterior gastric artery is a branch of the splenic artery and supplies the posterior stomach wall. For the first time it was mentioned by Walther in 1729 and after that rediscovered by Suzuki and coworkers in 1978. This artery can be found in 4 to 100%. Nevertheless, the authors report that it is present in 37.5-48% of cases [16, 17]. Okabayashi and coworkers report that computer tomography detects the posterior gastric artery in 98% and it usually begins from the splenic artery and rarely from the celiac trunk [18]. Two posterior gastric arteries to our knowledge were not previously reported in the literature.

Finally, there are incidental findings that developmental variations of vascular supply are linked to anomalies of the organs shape [19, 20]. Although case reports do not count as evidence-based medicine, they still represent valuable material for evaluation. Therefore, arterial variations should be studied in more detail taking into account organ morphology.

## 4. Conclusion

During the macroscopical dissection, we determined the accessory arteries of the upper abdominal cavity: left gastric, left hepatic, and two posterior gastric arteries, which have clinical importance in surgery. Textbook anatomy gives an overview of the most common type of developmental variation. Nevertheless, during interventional procedures one may encounter cases that are more complex. The presence of accessory gastric arteries may complicate gastric resections. Hepatic vascular anatomy is important during hepatic transplant and resections in case of pathological processes (for example, tumors). Finally, lesions to the posterior gastric arteries can lead to the necrosis of the posterior part of the stomach with drastic complications. Modern procedures in hepatopancreatobiliary and vascular surgery still depend on the knowledge of the regional anatomy and this case demonstrates there is much that can be learned from cadaver dissections.
